# Supplementary figures and images for: Generation and characterization of a tamoxifen-inducible, Cre driver rat for transgene expression in microglia
Source: Sci Rep. 2025 Dec 9;16:1579. doi: 10.1038/s41598-025-31077-z (PMC12800110; doi:10.1038/s41598-025-31077-z)

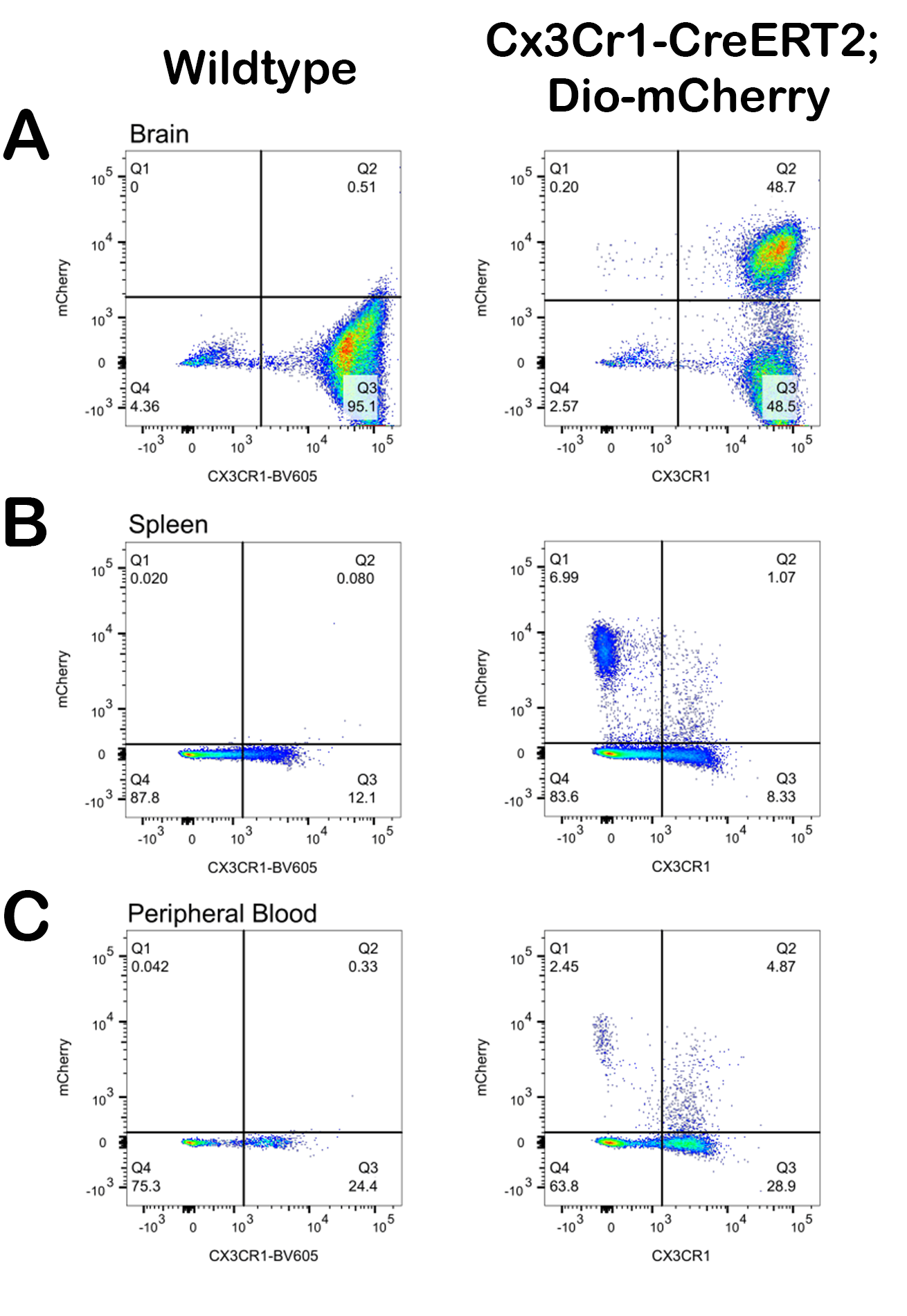

Supplement: Supplementary file 1 — Supplementary Material 1 [file 41598_2025_31077_MOESM1_ESM.png]

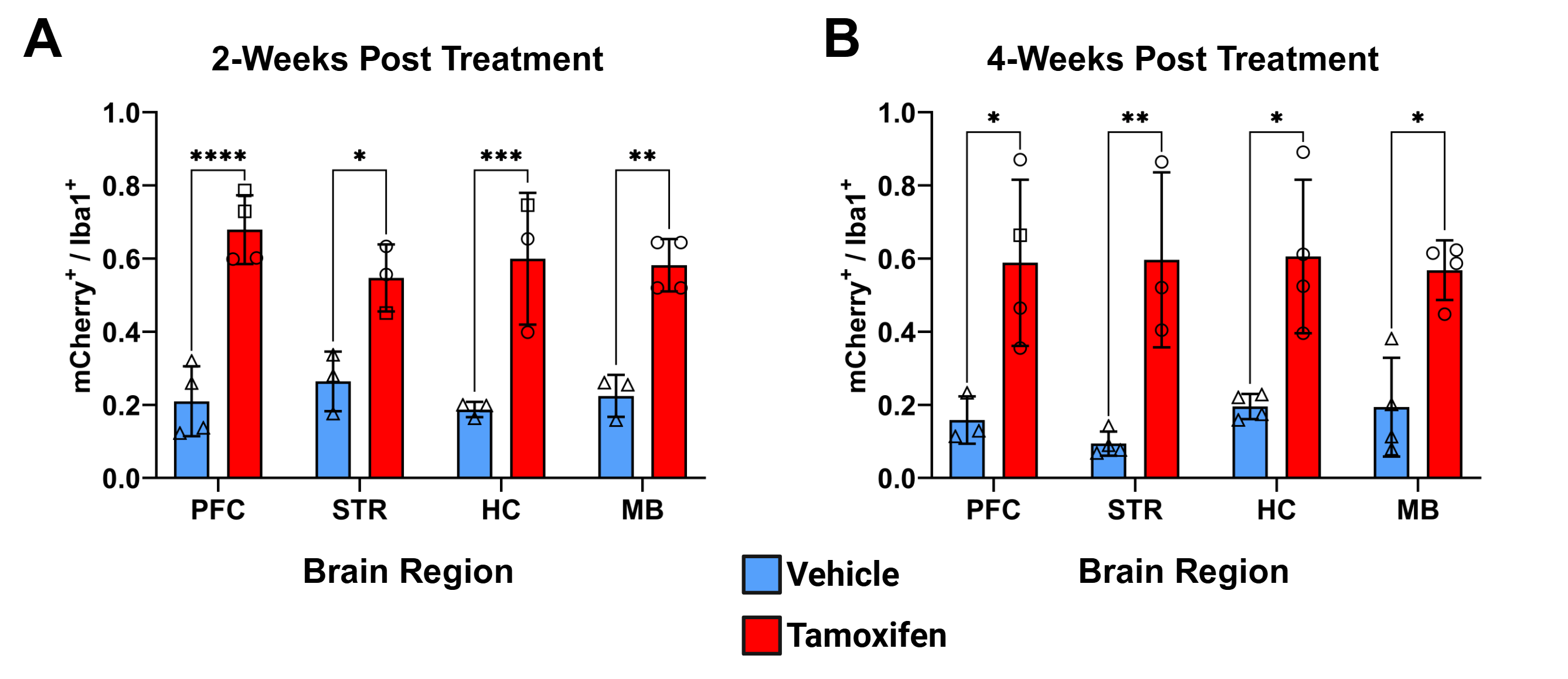

Supplement: Supplementary file 2 — Supplementary Material 2 [file 41598_2025_31077_MOESM2_ESM.png]

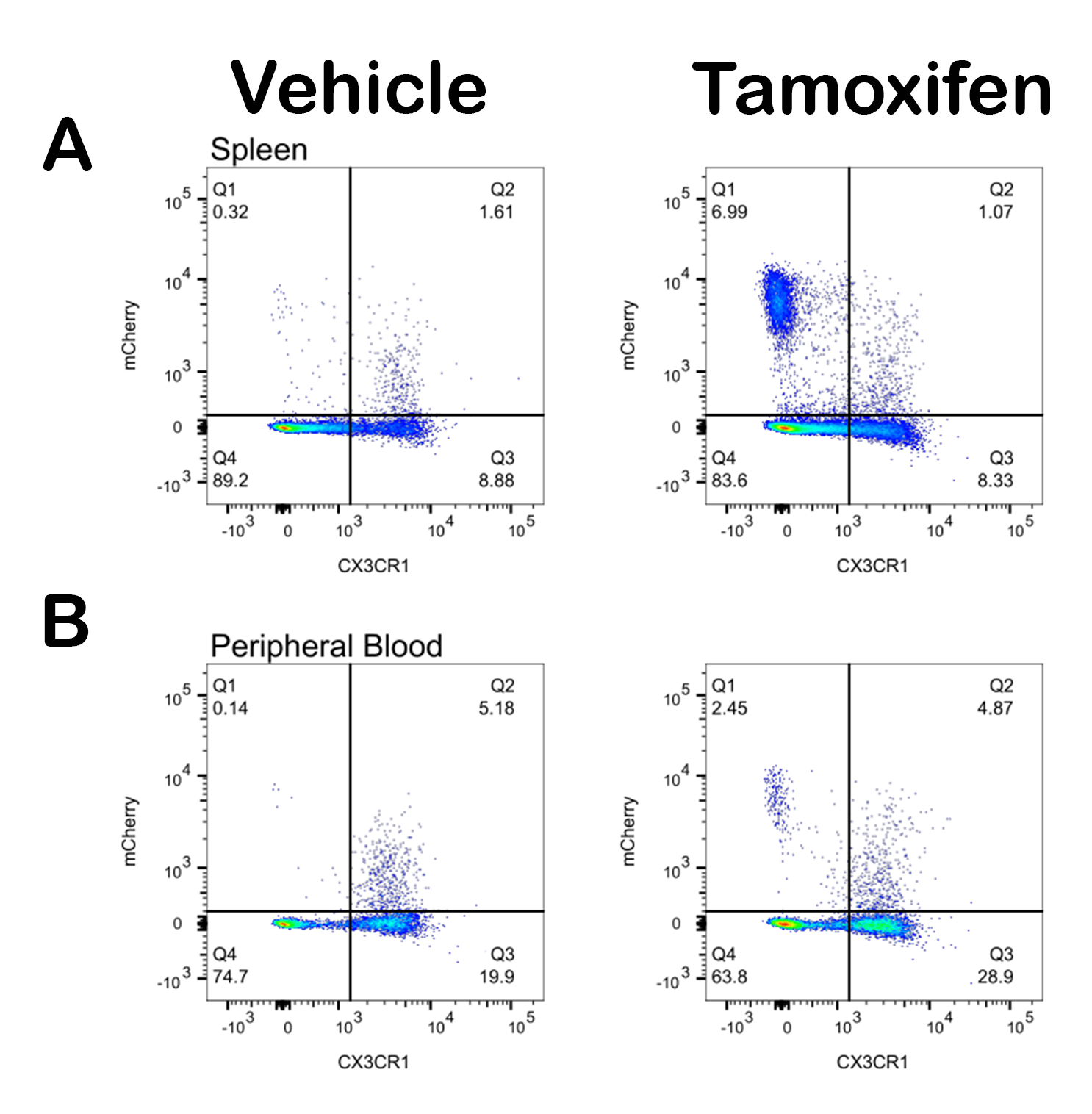

Supplement: Supplementary file 3 — Supplementary Material 3 [file 41598_2025_31077_MOESM3_ESM.png]

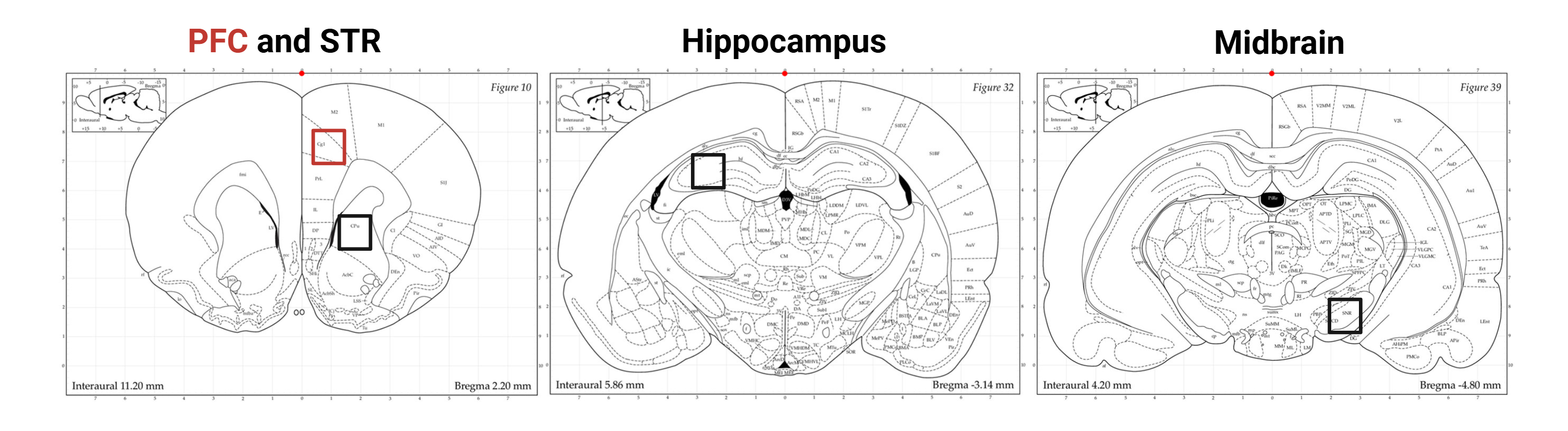

Supplement: Supplementary file 4 — Supplementary Material 4 [file 41598_2025_31077_MOESM4_ESM.png]

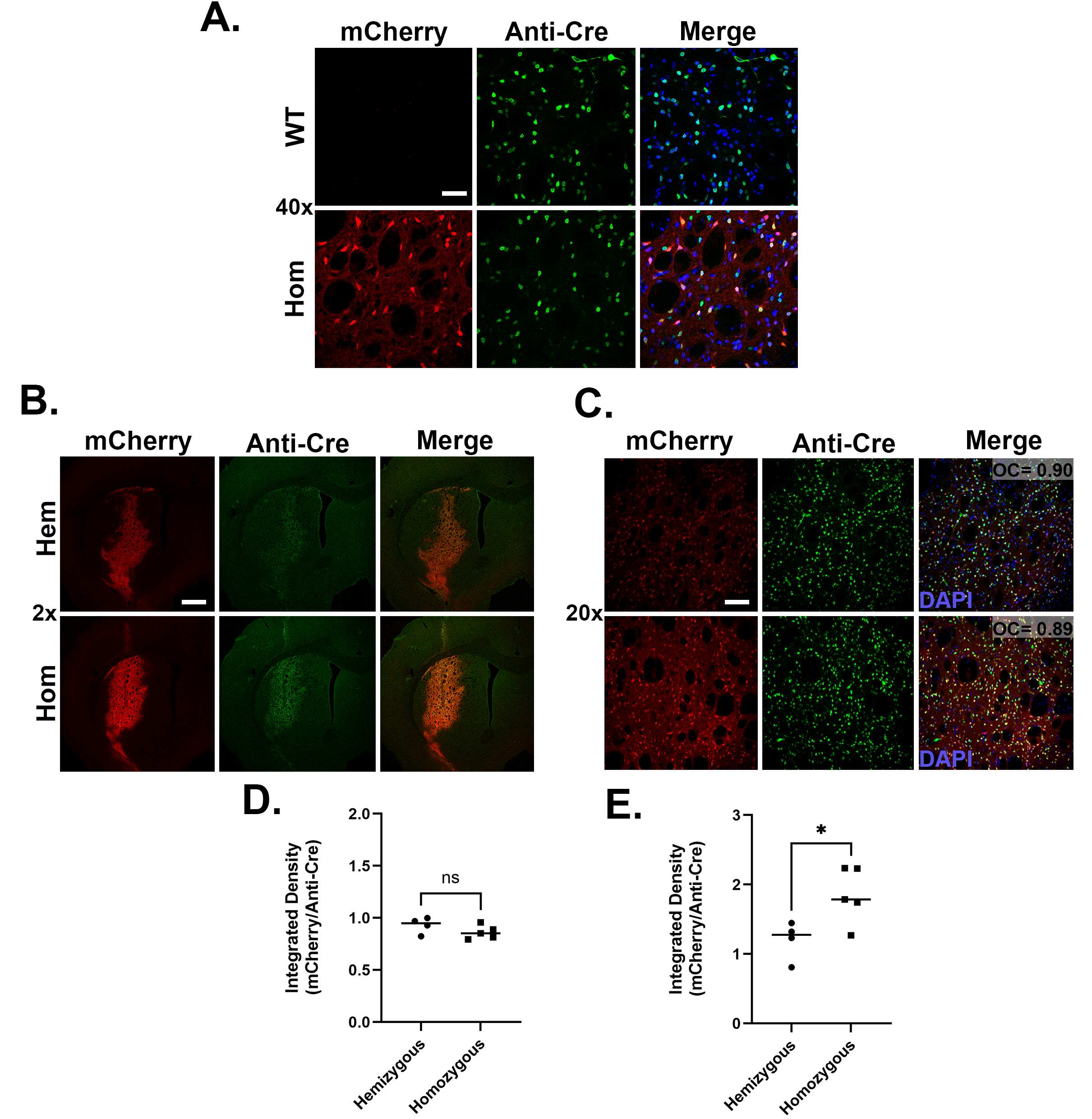

Supplement: Supplementary file 5 — Supplementary Material 5 [file 41598_2025_31077_MOESM5_ESM.png]

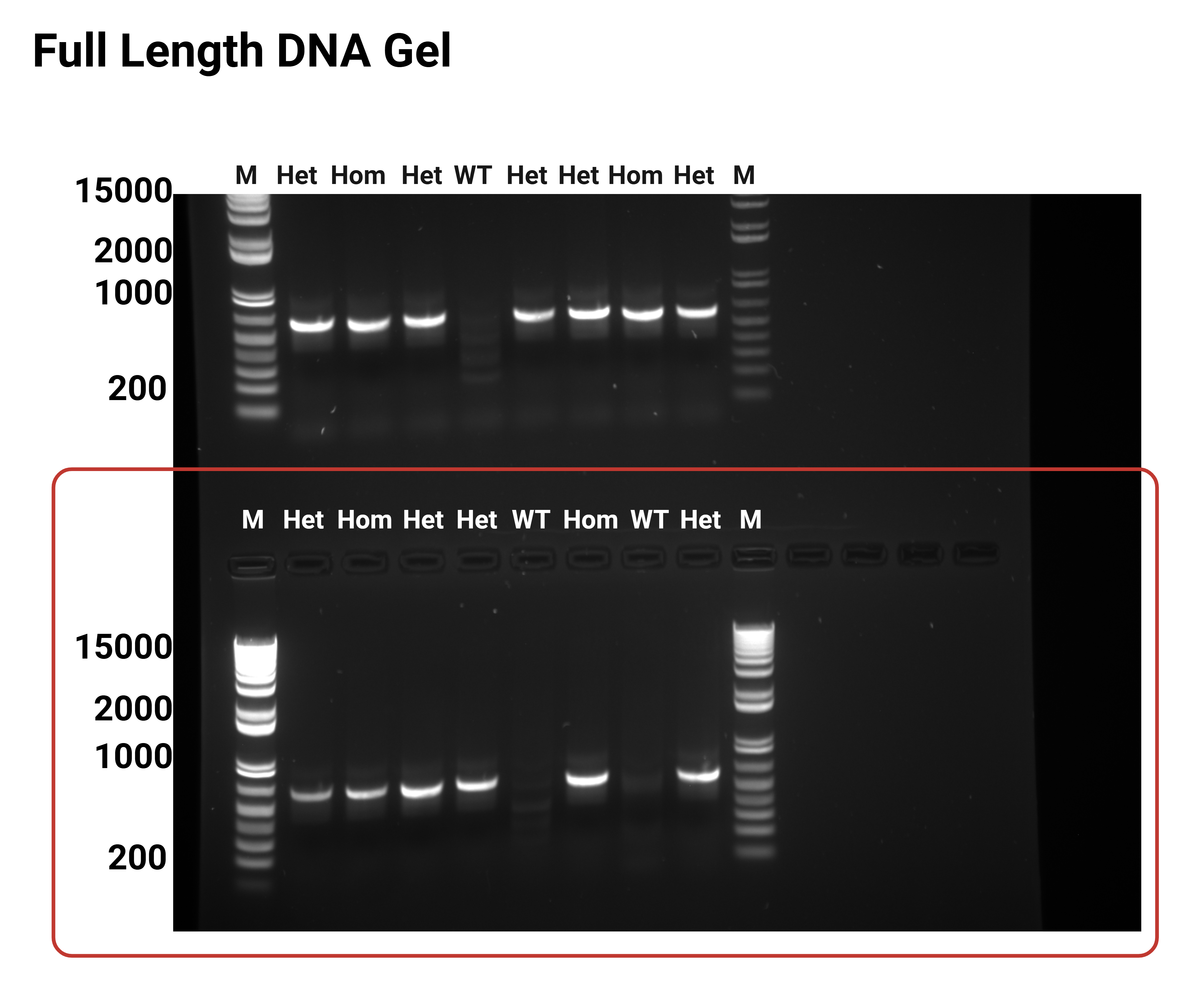

Supplement: Supplementary file 6 — Supplementary Material 6 [file 41598_2025_31077_MOESM6_ESM.png]

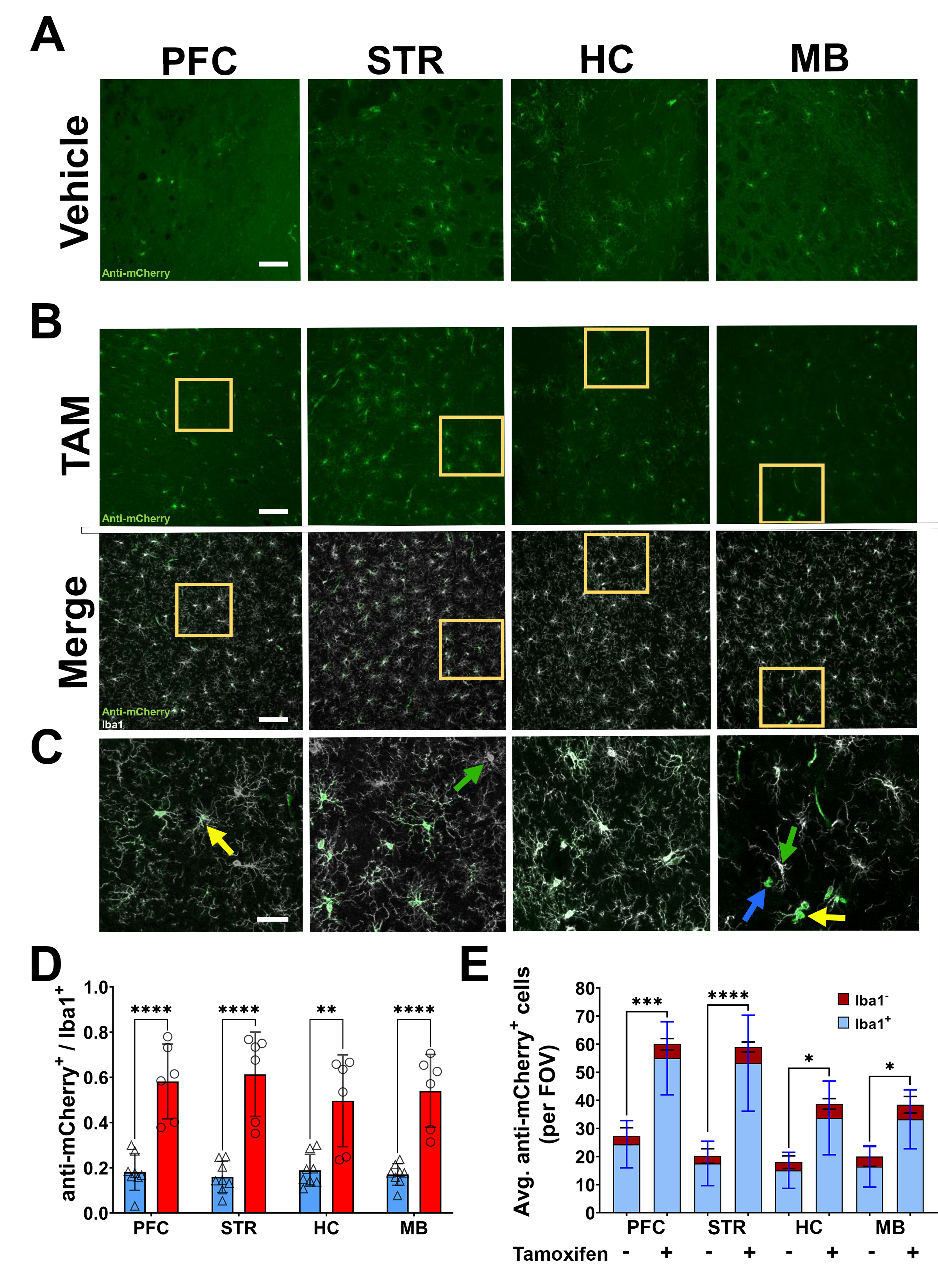

Supplement: Supplementary file 7 — Supplementary Material 7 [file 41598_2025_31077_MOESM7_ESM.png]

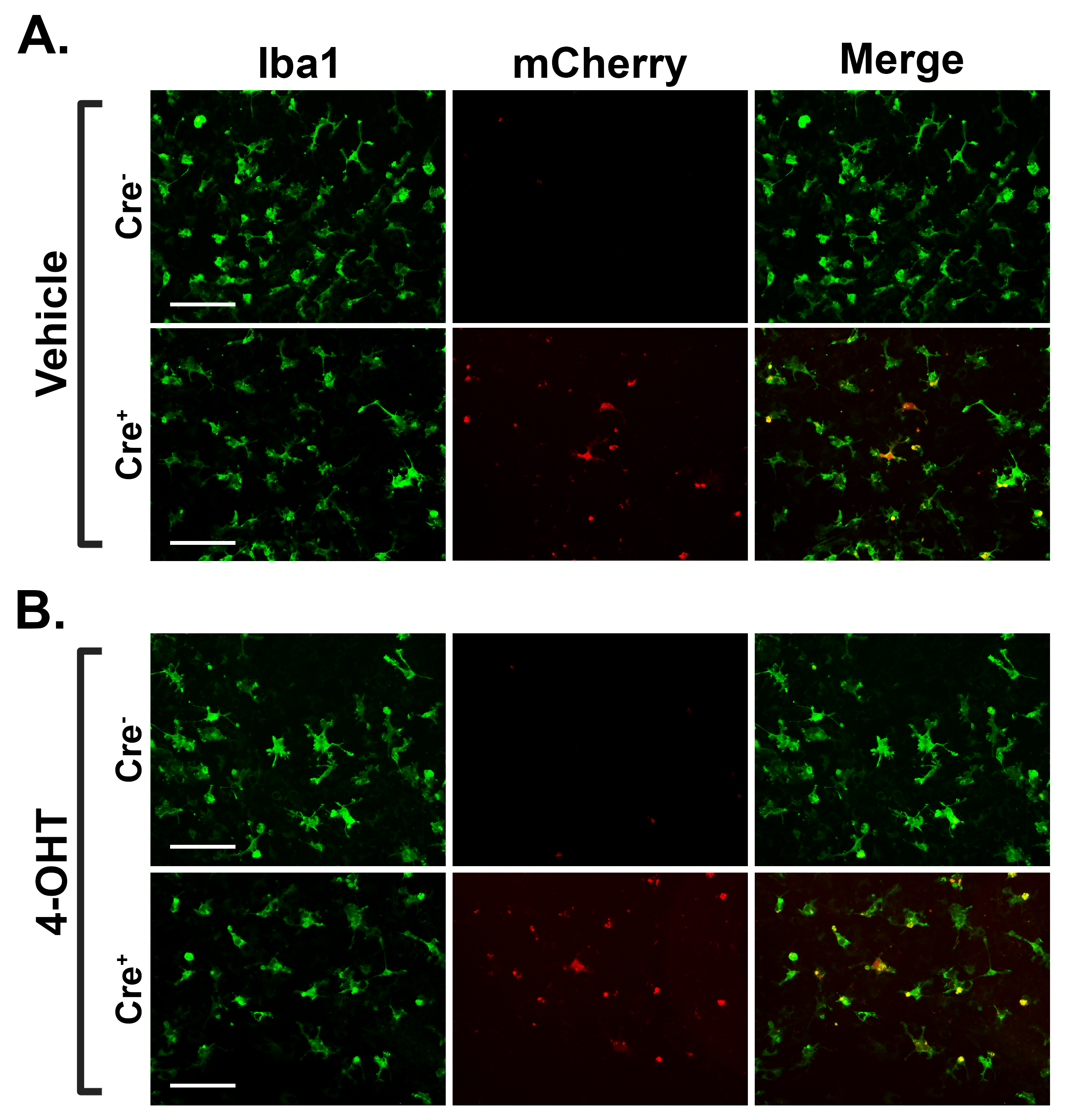

Supplement: Supplementary file 8 — Supplementary Material 8 [file 41598_2025_31077_MOESM8_ESM.png]

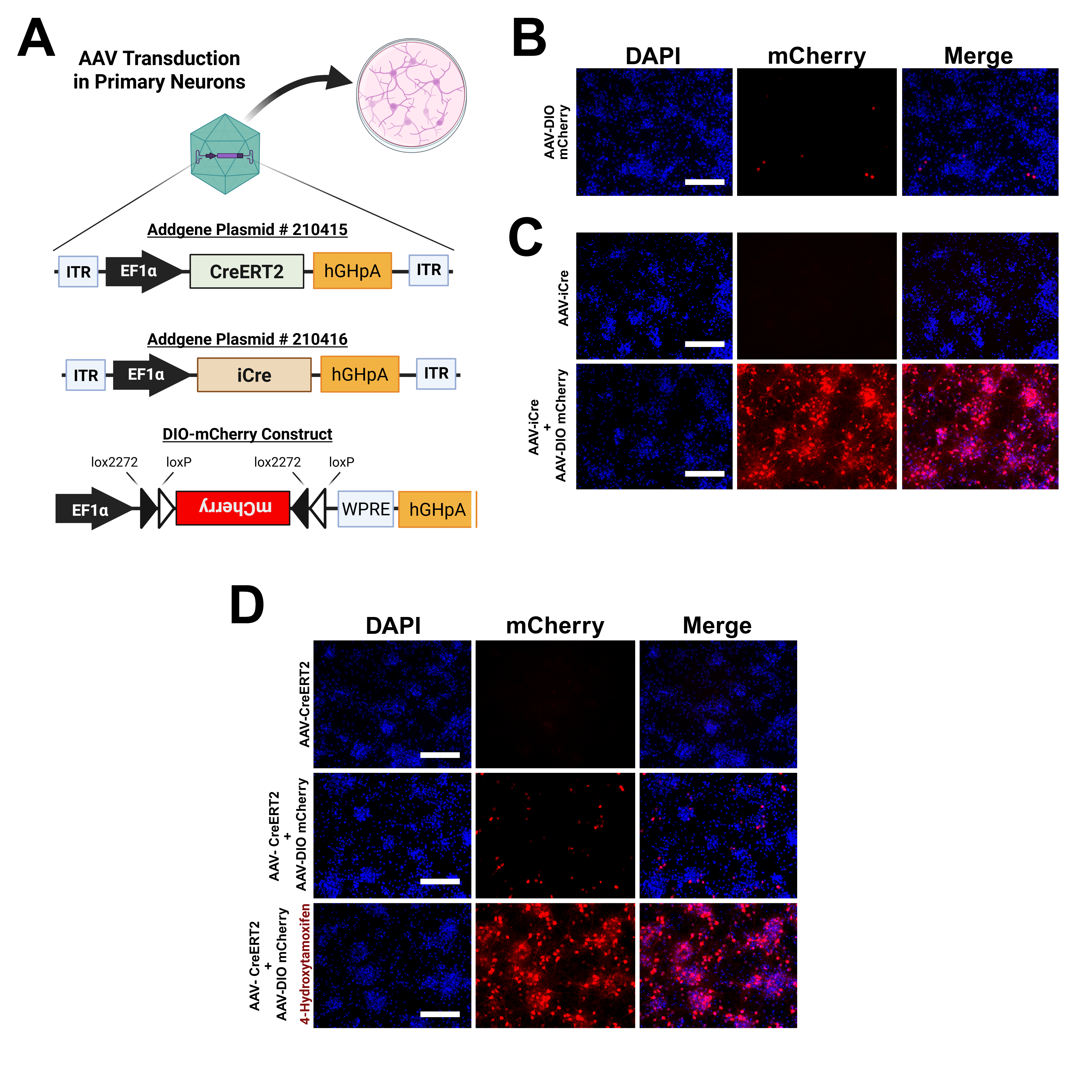

Supplement: Supplementary file 9 — Supplementary Material 9 [file 41598_2025_31077_MOESM9_ESM.png]

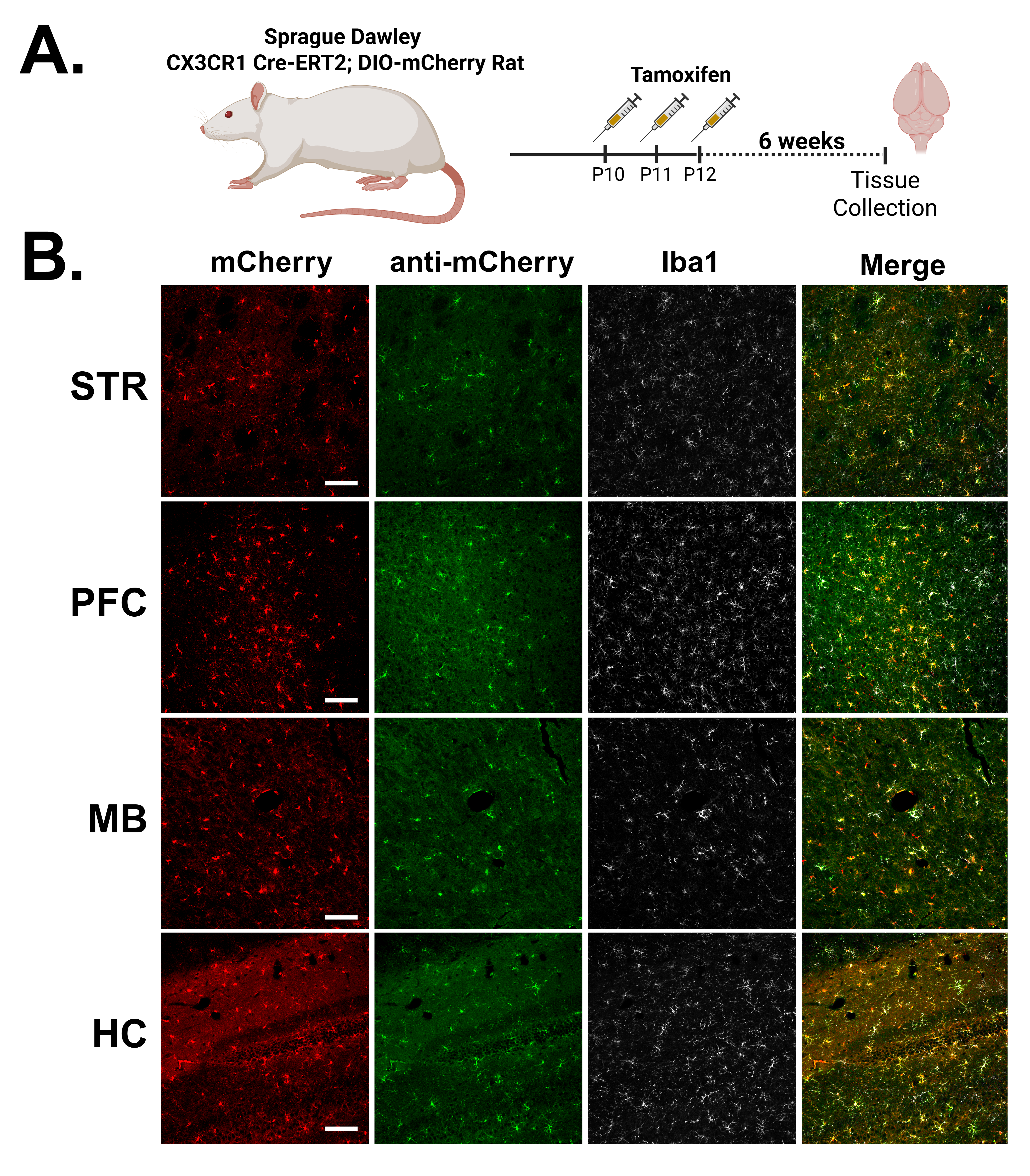

Supplement: Supplementary file 10 — Supplementary Material 10 [file 41598_2025_31077_MOESM10_ESM.png]
